# Necrotizing Cholecystitis in the Gallbladder: A Case Report

**DOI:** 10.7759/cureus.21368

**Published:** 2022-01-18

**Authors:** Tejaswita Katta, Khashayar Tavakoli

**Affiliations:** 1 Internal Medicine, Larkin Community Hospital, South Miami, USA; 2 Department of Surgery, State University of New York Downstate Medical Center, New York, USA

**Keywords:** candida, cholecystitis, cholecystectomy, necrotizing, abdominal pain

## Abstract

Infections caused by Candida species have shown a considerable increase in frequency in the recent past, and hence they are a cause of significant concern among medical practitioners. There are many factors that contribute to the occurrence of Candida-related infections in particular groups of patients. In this report, we present a case that highlights the causes and appropriate treatment methods of the condition. Patients with acute necrotizing cholecystitis often show poor outcomes after treatment, and hence physicians need to be alert when dealing with patients with this condition and should provide the best treatment method. We report a case of necrotizing cholecystitis in a 55-year-old female with a medical history of cholelithiasis, obesity, seizures, cocaine abuse, and anemia. She also reported lower abdominal pain, felt bloated, and complained of headache, dizziness, lack of appetite, shortness of breath, and vomiting. The patient underwent several lab tests as well as a CT scan of the abdomen, hepatobiliary iminodiacetic acid (HIDA) scan, endoscopy, and cholecystectomy.

## Introduction

Candida cholecystitis infections are often life-threatening complications. The risk of complications posed by Candida infection are similar to those due to risk factors such as cocaine abuse and cholelithiasis, and these include complex fistulae, antibacterial therapy, immunosuppression, prolonged stay in the ICU, and disseminated malignancy. Hence, maintaining a high index of early suspicion for the presence of this deadly fungal infection and administering aggressive surgical therapy are the only sure ways of attaining a favorable outcome [1]. Some studies indicate that diabetic individuals with low glycemic control are at high risk of Candida infections. In this regard, individuals hospitalized with diabetes are at risk of developing systemic infections caused by Candida famata, which culminates in gallbladder infections [2]. Some studies, however, dispute the hypothesis that candidiasis of the gallbladder is the common cause of acute cholecystitis and instead suggest that Candida cholecystitis is common among patients with malignancies. These conflicting positions about the causes of necrotizing cholecystitis emphasize the need for more studies to be conducted to help establish the cause and appropriate treatment of this condition.

## Case presentation

A 55-year-old female with a past medical history of cholelithiasis, hypertension (HTN), obesity, migraines seizures, cocaine abuse, bipolar disorder, and anemia initially presented with lower abdominal pain for a day. She rated the pain as 8/10 in intensity and also reported bloating, shortness of breath with inhalation, headache, and vomiting. She also reported feeling dizzy when standing up and stated that she had lost her appetite in the last three days. She, however, denied chest pains, nausea, and acute changes in hearing, photophobia, and dysuria. She also denied the use of any recreational drugs such as cocaine and marijuana as well as tobacco use.

**Figure 1:**
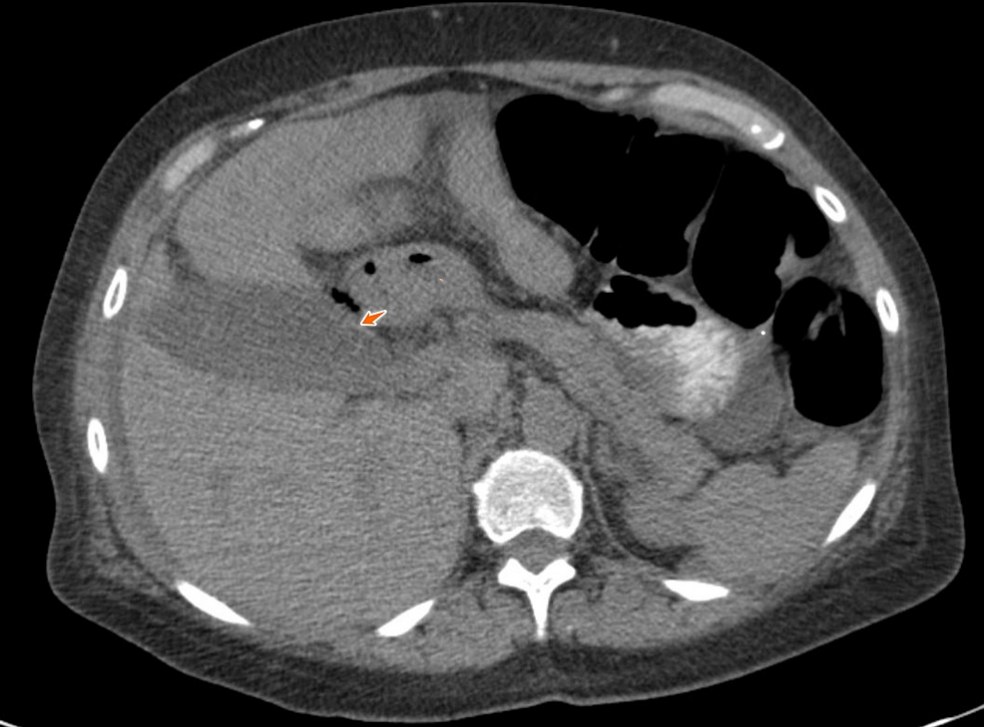
Gall bladder showing the presence of inflammation

**Figure 2:**
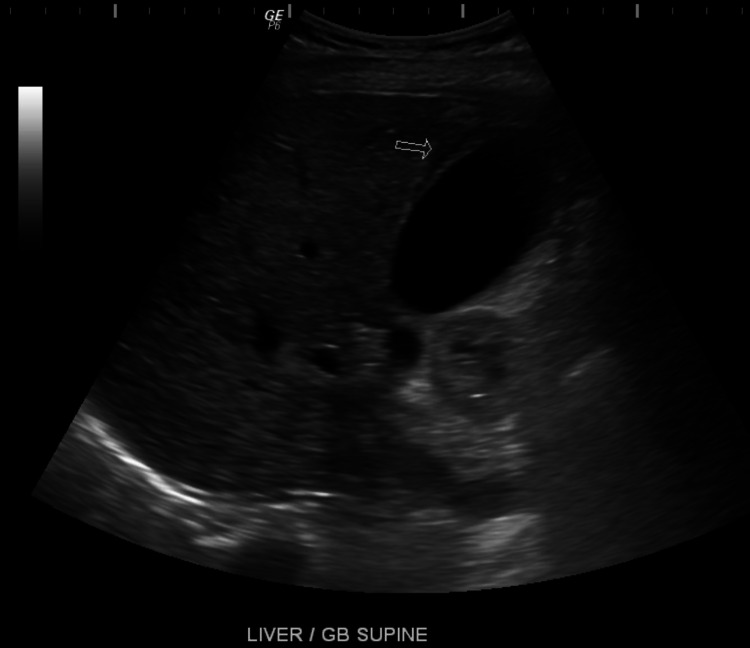
Gall bladder showing the presence of pericholecystic fluid

Upon examination, the patient was noted to have elevated levels of lipase (355 U/L), amylase (158 U/L), sodium of 132 mEq/L, elevated creatinine of 3.39 mg/dL, hemoglobin of 11.8 gm/dL, and mild leukocytosis at 11.8; her fecal occult blood test (FOBT) was negative. A CT of the abdomen was conducted and showed postsurgical changes consistent with a prior Roux-en-Y procedure. Interval development of distal small bowel wall thickening was observed, as well as a mild fat stranding and moderate pelvic and abdominal ascites that were significant on the right lower quadrant (Figure 1). The gallbladder showed interval development of layering densities and perihepatic ascites and an indication of a gallbladder sludge with cholelithiasis and hepatomegaly. Ultrasound of the gallbladder showed the presence of pericholecystic fluid that was secondary to the perihepatic ascites (Figure 2). The bladder and renal ultrasound revealed unremarkable results.

The patient was started on NS at 75; piperacillin/tazobactam and morphine were started, and a HIDA scan was done. The high levels of lactic acid and WBC supported the clinical diagnosis of systemic inflammatory response syndrome (SIRS). The patient’s past history of seizures prompted the consideration of drug-induced pancreatitis resulting from her home medication. Due to her previous Roux-en-Y anastomosis, recommendations to end further endoscopic procedures and endoscopic retrograde cholangiopancreatography (ERCP) were made.

**Figure 3:**
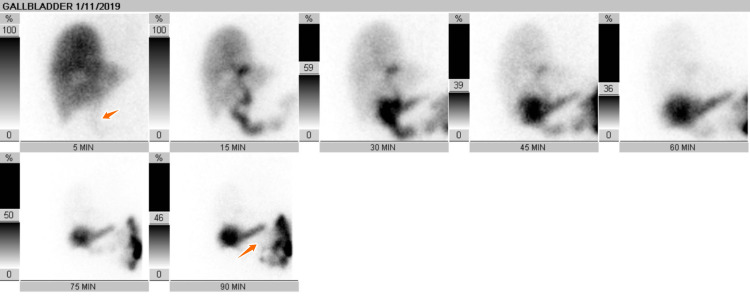
HIDA showing no biliary leak HIDA: hepatobiliary iminodiacetic acid

**Figure 4:**
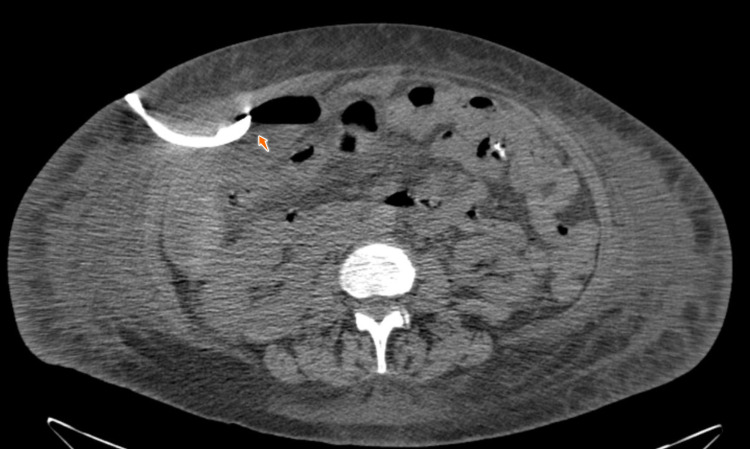
CT abdomen showing the presence of catheter for abscess drainage CT: computed tomography

On day three, lap cholecystectomy was conducted on the patient, and she showed no complications from the procedures for a few days. However, the WBC levels were found elevated at 26.6 three days after the cholecystectomy procedure. On day 13, a HIDA scan was performed after administering 5 mCi mebrofenin 99m technetium. The findings revealed no evidence of biloma and biliary leak (Figure 3). This was followed by a CT scan on day 14, which revealed fluid collection and mild ascites in the right perihepatic space (Figure 4). The abscess was drained using a catheter, and the patient's leukocytosis as well as her clinical status improved. Culture done on the fluid sample showed the presence of Candida. Examination of the gall bladder showed acute necrotizing cholecystitis with no gallstones identified.

## Discussion

According to Pappas et al. [3], Candida species are notorious for many fungal infections, ranging from non-life-threatening subcutaneous infections to invasive illnesses that attack various organs. Hence, infections resulting from Candida need a broad range of diagnostic procedures and appropriate therapeutic strategies.

In conditions such as acute cholecystitis, the culprit has always been Candida. However, according to Yildirim et al. [4], in over 90% of the patients diagnosed with acute cholecystitis, gallstones are often impacted in the cystic duct. However, in the presence of cholelithiasis and cholecystitis, several bacteria can be found in the walls of the gall bladder and bile. The organisms mostly found in the biliary tract include enteric Gram-positive bacteria such as Klebsiella, Enterobacter, Escherichia coli, and Proteus spp. Therefore, candidiasis of the gallbladder is not commonly associated with acute cholecystitis.

Laurila et al. [5] explain that acute acalculous cholecystitis is normally an inflammation of the gallbladder that is not caused by gallstones. The condition represents about 2-15% of all cases of acute cholecystitis and is often diagnosed in critically ill, postoperative, and trauma patients. Acute acalculous cholecystitis has an obscure clinical picture and its diagnostics is often tedious and difficult according to Prévôt et al. [6].

Some studies have also indicated that most necrotizing cholecystitis infections target elderly patients who exhibit age-related challenges in their biliary tract and have a history of frequent bacterial infections in their bile. The condition is often complicated in patients aged more than 70 years due to the presence of other conditions, including choledocholithiasis, acalculous cholecystitis, cancer, and gallbladder necrosis. This calls for earlier diagnosis and treatment as the condition tends to become more complicated with advancing age. Results from randomized controlled trials (RCTs) reveal that early surgery is recommended for necrotizing cholecystitis, usually seven days after the onset [7].

According to Inoue et al. [8], laparoscopic cholecystectomy (LC) is one of the standard treatment procedures for necrotizing cholecystitis. However, the application of this mode of treatment is difficult in patients with these conditions showing complications such as bile duct injury, bile leak, and bowery injuries. Therefore, it is important for physicians to thoroughly evaluate the severity of the inflammation caused by the condition so that the most appropriate treatment method can be selected. In 2007, the Tokyo Guidelines were issued and internationally accepted as the standard for diagnosing and treating cholecystitis. According to these guidelines, the criteria for treatment should be based on clinical symptoms, blood tests, physical examinations, and imaging findings. The results should then be used to classify the extent of inflammation into three grades: grade I (mild), grade II (moderate), and grade III (severe). The appropriateness of the therapy, therefore, is determined by the grade of the infection. The guidelines recommend that grade I and II patients undergo immediate LC while grade III patients should strictly undergo percutaneous transhepatic gallbladder drainage (PTGBD).

## Conclusions

We reported a case of necrotizing cholecystitis caused by Candida in a 55-year old female who was not diabetic and had no malignancy. Our primary objective was to show that this condition is caused by various factors, and hence there is a need for proper diagnosis and administration of proper treatment. We recommend that efforts be taken to raise awareness on the impact of Candida on necrotizing soft tissues by illustrating its features and outlining the course of diagnosis and treatment that should be taken up as standard care.
